# Early cost-effectiveness analysis of a novel rapid diagnostic test for tuberculosis in rural Philippine settings

**DOI:** 10.1371/journal.pgph.0005364

**Published:** 2025-10-28

**Authors:** Aren Sargood, Abigail Ortal-Cruz, Rusheng Chew, Chris Painter

**Affiliations:** 1 Oriel College, University of Oxford, Oxford, United Kingdom; 2 San Lazaro Hospital–Nagasaki University Collaborative Research Office, Manila, Philippines; 3 Mahidol-Oxford Tropical Medicine Research Unit, Bangkok, Thailand; 4 Nuffield Department of Medicine, Centre for Tropical Medicine and Global Health, University of Oxford, Oxford, United Kingdom; 5 Lao-Oxford-Mahosot Hospital-Welcome Trust Research Unit (LOMWRU), Mahosot Hospital, Vientiane, Lao PDR; University of Sydney, AUSTRALIA

## Abstract

Tuberculosis disproportionately affects low- and middle-income countries (LMICs), where gold standard molecular diagnostic assays like Xpert MTB/RIF are relatively frequently inaccessible. A novel rapid molecular diagnostic test (nLRDT), which can test tongue swab samples, and is more suitable for decentralised primary healthcare settings, has been developed and is currently undergoing preclinical validation. This study evaluated the early cost-effectiveness of this nLRDT vs. Xpert MTB/RIF for diagnosing pulmonary tuberculosis in rural Philippine primary healthcare settings. A hybrid decision tree and Markov model-based cost-effectiveness analysis was conducted from health provider and societal perspectives. Parameters were taken from relevant literature, national-level data, and expert opinion. Costs were expressed in 2024 US dollars and cost-effectiveness evaluated by comparing incremental cost-effectiveness ratios with willingness-to-pay threshold estimates. An estimated willingness-to-pay (WTP) threshold of $1,357 per quality-adjusted life year (QALY) was used. The nLRDT was cost-effective with sputum samples, from both perspectives, with net monetary benefits (NMBs) of $26.62 (societal perspective) and $19.21 (provider perspective) at the WTP threshold of $1,357. Similarly, the nLRDT was estimated to be cost-effective for tongue swab samples from both perspectives, with NMBs of $43.81 (societal) and $24.79 (provider). Sensitivity and scenario analyses identified the specificity of nLRDT as the key driver of cost-effectiveness, with cost-effectiveness maintained at greater WTP thresholds and varying combinations of test performance characteristics. Our findings suggest the nLRDT can be a cost-effective diagnostic tool in rural Philippines, and possibly other similar, contexts. This has positive research, industry, and policymaking implications.

## Background

Tuberculosis (TB) is an airborne disease caused by the bacterium *Mycobacterium tuberculosis* (MTB) [1]. Pulmonary tuberculosis (PTB) is the most prevalent form of the disease [2]. In 2022, there were an estimated 10.6 million TB cases and 1.3 million deaths worldwide [3]. Multidrug-resistant TB (MDR-TB) strains, which do not respond to first-line drugs rifampicin and isoniazid [4], are more difficult to treat than drug-sensitive tuberculosis (DS-TB) [5]. TB disproportionately affects low-income populations, where overcrowding raises infection risk for household contacts of infected individuals. Malnutrition and diabetes worsen outcomes for TB patients, which are both more prevalent among low-income populations [6]. Over 95% of TB cases and deaths occur in low- and middle-income countries (LMICs) [7]. In 2022, 80% of TB services expenditure came from domestic sources, highlighting a disproportionate financial strain on patients living in LMICs [3].

The gold standard for TB diagnosis is culture, but this method takes weeks to yield results [8]. Consequently, sputum smear microscopy, despite its low sensitivity, remains the mainstay diagnostic tool in many LMICs [8]. The World Health Organization (WHO) End-TB strategy targets 80% and 90% reductions in TB cases and deaths, respectively, between 2015 and 2030 [9]. Achieving these requires adopting WHO-recommended molecular tests [10], like the Xpert MTB/RIF assay (Cepheid, Sunnyvale, USA) (‘Xpert’), which offers high sensitivity and specificity, and detects MTB and rifampicin resistance (RR) in approximately two hours [11]. Global molecular test use increased from 38% in 2021 to 47% in 2022 [12], significantly below the United Nations target of 100% by 2027 [13], largely due to high costs [14]. Xpert requires skilled personnel, controlled temperature, stable electricity, and sophisticated machines and cartridges, typically limiting its availability to urban, centralised settings [14]. Additionally, Xpert requires sputum samples to diagnose pulmonary TB. Some patient groups, e.g., young children, often struggle with sputum collection, necessitating invasive procedures such as nasogastric aspiration [15]. The WHO’s Target Product Profile (TPP) for TB diagnostics seeks a rapid, point-of-care molecular test that processes non-sputum samples, requires minimal training, and operates without complex equipment or electricity, making it suitable for decentralised healthcare settings, enhancing TB care access [16].

A novel rapid molecular test utilising loop-mediated isothermal amplification (LAMP) coupled with lateral flow dipstick (LFD) has been developed. The wet format demonstrated 100% specificity and sensitivity in sputum, in vitro, with a turn-around time of approximately one hour [17]. Having been converted into a dry format test, with results displayed on a lateral flow test strip, it is currently undergoing preclinical validation in the Philippines. This novel LAMP-based rapid diagnostic test (nLRDT) is able to use tongue swab samples as a test substrate; however, it does not test for resistance. The test eliminates the need for sputum transportation, and does not require highly-trained staff or complex equipment. Furthermore, 16–20 samples can be processed using nLRDT in the same two-hour period that Xpert can process 4 samples [18], and it is likely to be more affordable than its competitors. These characteristics suggest that nLRDT is highly suitable for use by rural, low-skilled workers and has the potential to meet the WHO TPP, provided that ongoing field studies confirm sufficient sensitivity and specificity for both sputum and tongue-swab samples.

Evaluating the nLRDT’s potential cost-effectiveness and performing uncertainty analyses to understand the key drivers of cost-effectiveness will inform progression along the translational research pathway and ensure the right data are collected in large-scale clinical trials. Accordingly, the study aims to assess the cost-effectiveness of nLRDT incorporation into the clinical pathway of presumptive PTB patients in a high-burden LMIC setting, the Philippines.

## Methods

### Setting

The Philippines, an LMIC with 119 million people and a median age of 25 [19], has over 50% of its population living in rural areas. Furthermore, 36% of the rural population live in poverty, compared with 13% in urban settings [20]. It is classified by the WHO as a “high TB burden” and “high MDR/Rifampicin Resistant (RR)-TB” country [21]. In 2022, there were an estimated 737,000 new TB cases, constituting 7% of global cases, with 4.2% being MDR/RR-TB, and a case-fatality ratio of 6% [22].

At the primary healthcare (PHC) level, TB care is delivered through a decentralised public network of health centres (HCs), barangay health stations (BHSs), and rural health units (RHUs) [23]. Xpert has been the standard diagnostic technique for all presumptive TB cases since 2019 [24], though its implementation is still being scaled up [25]. In some rural areas, sputum smear microscopy remains in use [25]. Xpert is generally unavailable at HCs, BHS, and RHUs, where initial TB care is typically sought [23]. Sputum samples are collected at these sites and transported to laboratories in urban centres for testing [23], with one Xpert machine often shared by 10 municipalities [25]. The logistical challenges of transporting sputum samples are significant, especially given the country’s 7,000 islands, disproportionately affecting rural and remote areas [25]. PhilHealth, the national health insurance scheme, has covered TB-related costs since 1995, with over 90% coverage in 2019 [26].

The Philippines was chosen as it represents an ideal target setting for an improved and cheaper diagnostic technique, due to high TB prevalence, decentralised clinics with limited on-site diagnostic capacity, and insufficient resources to fund Xpert widely [23]. Additionally, this setting provides an opportunity to evaluate the nLRDT cost-effectiveness in a ‘high resistance’ environment, given its inability to test for RR.

### Model design and participants

A model-based cost-effectiveness analysis was conducted to compare the current standard of care for diagnosing presumptive PTB in the Philippines with a hypothetical pathway involving the nLRDT as the initial intervention. Model participants were individuals presenting to rural primary healthcare centres with symptoms suspicious for PTB, defined according to the National TB Program Manual of Procedures 6th edition (NTPMP6) as having either (i) ≥2 weeks of: cough, unexplained fever, unexplained weight loss, or night sweats, or (ii) a chest X-ray finding suggestive of TB.

### Model structure

**Fig 1:**
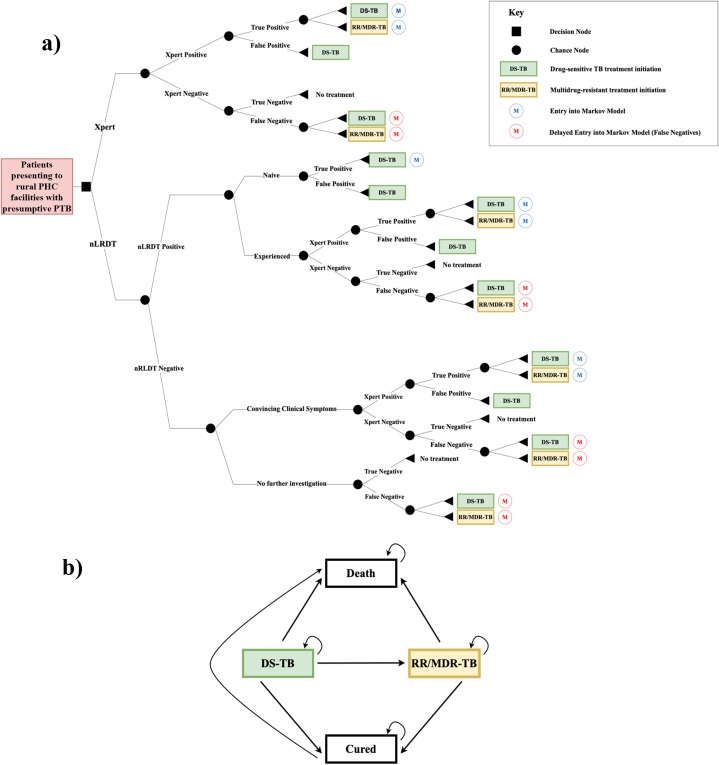
(a) Decision tree model structure (b) Markov model structure. Abbreviations: DS-TB, drug sensitive tuberculosis; MDR-TB, multidrug-resistant tuberculosis; PTB, pulmonary tuberculosis; MTB, Mycobacterium tuberculosis; RR, rifampicin resistance.

The model structure development was aided by targeted literature searches of PubMed, focusing on studies using keywords such as “cost-effectiveness analysis”, “suspected tuberculosis” and “GeneXpert”. The structure was substantially informed by Chitpim et al. [27], which evaluated costs and outcomes of five TB diagnostic algorithms (Xpert included) in Thailand, using a decision tree and Markov model hybrid model. The decision tree effectively modelled the set of testing strategies, capturing associated costs, whilst the Markov model reflected the lifelong impacts of treatments for different patient cohorts following diagnosis. This approach facilitates a comparison of the costs and benefits of integrating the nLRDT into the clinical pathway versus the current standard of care in the Philippines, thereby justifying its selection. The structure was also informed by a theoretically practical and contextualised integration of the nLRDT provided by the second author, a physician based in San Lazaro Hospital, Manila – a ‘Special National Hospital Medical Center for Infectious Diseases’ [28], following careful consideration of the current diagnostic pathway and infrastructure. Fig 1 depicts the model structure, with two comparative diagnostic approaches:

Under the current diagnostic strategy (Xpert branch from decision node), all patients underwent Xpert testing for MTB and RR. Positive MTB results indicated either true or false positives, with subsequent consideration of the resistance result. Negative MTB results, precluding a resistance result, indicated either true or false negatives. True negatives were treated as part of the general population, whereas false negatives were assumed to undergo further investigation upon symptoms worsening. The proposed strategy (nLRDT branch from decision node) involved initial MTB testing with the nLRDT. nLRDT-positive patients were categorised based on their TB treatment history, due to significantly higher resistance rates among treatment-experienced patients. Whilst it was assumed that there was no resistance present among treatment-naïve patients due to very low (1.8%) resistance rates in this cohort [29]. Treatment-naïve patients began DS-TB treatment and were either true or false positives. Treatment-experienced patients undergo a further Xpert test to determine resistance. As Xpert is a WHO-endorsed molecular test, the result superseded the nLRDT result. A proportion of nLRDT-negative patients with convincing symptoms underwent a further Xpert test at the physician’s discretion, with Xpert results taking precedence. The remaining nLRDT-negatives represented either true or false negatives.

Following diagnosis, true positives and false negatives (i.e., patients who actually had PTB) entered the Markov model. The four mutually exclusive health states were:

i DS-TB Treatment,
ii MDR-TB Treatment,
iii Cured,
iv Dead.

Patients entered the Markov model at states (i) or (ii). A 1-month cycle length was adopted, consistent with the methodology of Chitpim et al. [27]. Treatment outcome data, which is based on regimen length, were converted to monthly (one cycle) transition probabilities using the equation outlined in Gidwani et al. [30]. Unlike Chitpim et al. [27], it was assumed there was no extensively drug-resistant TB (XDR-TB) due to the 0% rate found among Xpert MTB-positive patients in the 2018 Philippine Drug Resistance Survey [29]. As the focus of the model was to compare diagnostic interventions, the treatment component was modelled pragmatically.

It was assumed there were no re-infections, so ‘cured’ patients remained cured for life. Patients failing DS-TB treatment began second-line MDR-TB treatment, and those failing MDR-TB treatment were assumed to die. Natural death rates were incorporated using life expectancy tables for the Philippines [31]. A lifetime time horizon was analysed and the model continued until all patients had died.

Outcomes of interest were quality-adjusted life years (QALYs) and total costs. Cost-effectiveness was evaluated using incremental cost-effectiveness ratios (ICERs). As the Philippines does not use an explicit willingness-to-pay (WTP) threshold, an estimate of $1357/QALY, derived from a 2023 study [32], was utilised to aid result interpretation. A 7% annual discount rate on costs and QALYs was applied, as recommended by the Philippines’ Health Technology Assessment Methods Guide [33], with no age-weighting, following Chew et al. [34].

Two perspectives were adopted: 1) healthcare provider and 2) societal. The societal perspective incorporated patient’s out of pocket costs and productivity losses as well as the healthcare provider’s costs. The human capital approach was used to estimate productivity losses. The societal perspective was chosen to reflect the significant patient financial burden and treatment cost differences between DS-TB and MDR-TB. Studies estimate mean total costs of $601.40 for TB-affected households, equating to 2.4 times pre-diagnosis monthly income, with MDR-TB household costs ($2,919.50) vastly greater than DS-TB ($562.40) [26].

The modelling process used the ‘rdecision’ v1.2.0 package in R [35]. Due to limitations with this package, the Markov model outputs (costs and QALYs) could not be directly integrated into the decision tree model. Consequently, the Markov model outputs were entered as outcomes of the decision tree with associated uncertainty distributions to reflect uncertainty in the Markov model. The analysis was performed using R (R Foundation for Statistical Computing, Vienna, Austria).

### Model approach

Several assumptions were made in the analyses, these are detailed thoroughly in Table A in S1 File. Table 1 shows the assumed schedule for the different tests that a patient entering the decision tree can experience. These were used to calculate provider and patient costs of tests.

**Table 1:** The assumed schedule of tests that a patient entering the decision tree can experience.

**Initial Xpert Test (including false negatives) (3-day visit):** Day 1: Initial consultation (diagnostic meeting) Day 2: Sputum collection (pre 9am) (+ shipping + Xpert test) Day 3: Results & treatment consultation
**Initial nLRDT Test (2-day visit):** Day 1: Initial consulation (diagnostic meeting) Day 2: Sputum collection + results & treatment consultation
**Further Xpert Test after initial nLRDT test (2-day visit):** Day 1: Sputum collection (pre 9am) (+ shipping + Xpert test) Day 2: Results & treatment consultation

To calculate the treatment outcome probabilities from WHO data for the Philippines [36,37], ‘lost to follow up’ and ‘not evaluated’ cases were omitted. The probability of false negatives dying was increased by 50% against the true positive probability due to delayed treatment initiation, informed by a study from Southwest Ethiopia showing treatment deaths more than doubled in patients with treatment delays over 30 days [38]. This conservative adjustment accounts for the Philippines’ higher healthcare spending as a share of GDP compared to Ethiopia [39].

Standard uncertainty distribution assumptions were applied to all model parameters (except Xpert sensitivity and specificity which were obtained from the literature). These were beta distributions for proportions and gamma distributions for all costs and QALYs, using a 10% standard deviation on the mean, following Chew et al. [34]. All parameter calculations were performed in Excel (Microsoft, Washington, USA). A detailed list of the model parameters and the associated uncertainty distributions can be found in the Table B in S1 File.

### Scenario analysis

Two scenarios were explored. Scenario 1 (the standard scenario, assumed unless otherwise specified) adheres to the outlined assumptions. Scenario 2 models the scenario where the nLRDT processes tongue swab samples, enabling completion of the diagnostic process within one day (tongue swab can be taken on same day as initial presentation) and eliminating sputum collection costs. Within each scenario, six combinations of nLRDT sensitivity and specificity are evaluated:

- Four conservative test performance scenarios (C1 - C4) where the sum of sensitivity and specificity equals 150%; the ‘rule of thumb’ minimum value for a test to be considered useful [40]. This follows the approach of Chew et al. [34].
- A base case. The base case for the standard scenario was 80% sensitivity and 98% specificity. The base case for the tongue swab scenario assumes lower sensitivity, as indicated by Andama et al. [41], who found reduced sensitivity in another molecular test (Xpert MTB/RIF Ultra) when conducted on tongue swabs; therefore, values of 70% for sensitivity and 98% for specificity were assumed.
- A high test performance scenario – 90% sensitivity & 98% specificity for both scenarios.

A wide range of values were considered due to the reported importance of test accuracy on cost-effectiveness results for comparator diagnostics [42], as well as clinical accuracy of the nLRDT not yet being established. Furthermore, accounting for large-scale deviations from expected outputs provides decision-makers with good boundaries of the outcome to better interpret the data for informed policymaking [43].

### Uncertainty analysis

Univariate deterministic sensitivity analysis (UVA) assesses the impact of individually varying each parameter on ICERs, using the 2.5th and 97.5th percentiles of the parameter’s distribution as lower and upper estimates. This approach identifies which parameters’ uncertainties most heavily impact cost-effectiveness. Since the ‘QALY’ parameters (e.g., DS-TB QALYs and false negative DS-TB QALYs) are, in reality, not independent, the analysis simultaneously adjusted all four QALY-related parameters to both their lower and upper estimates, creating a new parameter, ‘Model QALYs’, to represent this. Results were presented in a tornado diagram for the eight most significant parameters by absolute change in net monetary benefit (NMB).

A multivariate deterministic sensitivity analysis (MVA) was conducted to explore how interactions between key variables impact the estimated NMB of the nLRDT. Key parameters (TB prevalence, nLRDT sensitivity and specificity, and the proportion of treatment naive patients) were chosen due to their impact on model outcomes, alongside their inherent uncertainty. These were varied simultaneously within realistic ranges to capture nonlinear relationships that UVA fails to capture, providing pragmatic results, given multiple factors will vary together in real-world scenarios [44].

A probabilistic sensitivity analysis (PSA) was performed using 1000 Monte Carlo simulations on base case (standard scenario) assumptions. Results, from both societal and provider perspectives, were presented on a cost-effectiveness plane. PSA was performed as it is the only sensitivity analysis that can address all uncertainties in all model parameters simultaneously.

The report for this study was produced in accordance with the Consolidated Health Economic Evaluation Reporting Standards (CHEERS) guideline [45].

## Findings

### Scenario analysis

Table 2 illustrates that in the base case the nLRDT is cost-effective in both scenarios and from both perspectives. It is important to note that the results for the considered scenarios span all four quadrants of the cost-effectiveness plane, and as such the interpretation of the ICER values against the WTP threshold changes between the north-east and south-west quadrants. In the north-east quadrant, ICER values below the WTP threshold indicate the nLRDT is cost-effective, whereas in the south-west quadrant ICER values *greater* than the WTP threshold indicate the nLRDT is cost-effective. The NMB results are presented for easier interpretation, where positive values indicate cost-effectiveness regardless of position on the cost-effectiveness plane. Threshold cost-effective prices considerably exceed the estimated nLRDT cost of $10 in all four of the base case results. Additionally, the nLRDT is more cost-effective in the tongue swab base case than in the standard scenario, from both perspectives, despite the slightly lower assumed sensitivity of using tongue swab samples. In all conservative test performance cases, the nLRDT yields a higher NMB in the provider perspective than in the societal perspective. Conversely, with improved test performance in the base and high-performance cases, the nLRDT yields a higher NMB in the societal perspective than the provider perspective.

**Table 2:** Effect of varying sensitivity and specificity of the nLRDT for Scenario 1 and Scenario 2.

	Scenario 1: Standard Scenario									
			**Societal Perspective**				**Provider Perspective**			
	**nLRDT Sensitivity, %**	**nLRDT Specificity, %**	**ICER (nLRDT vs. Xpert)**	**Cost-Effectiveness Plane Quadrant**	**NMB, US$**	**Threshold cost-effective price, US$**	**ICER (nLRDT vs. Xpert)**	**Cost-Effectiveness Plane Quadrant**	**NMB, US$**	**Threshold cost-effective price, US$**
**C1**	**60**	**90**	nLRDT Dominated	NW	-8.59	1.41	4,601.07	SW	**10.77**	20.77
**C2**	**70**	**80**	nLRDT Dominated	NW	-46.68	-36.68	4,325.43	SW	**6.16**	16.16
**C3**	**80**	**70**	nLRDT Dominated	NW	-84.77	-74.77	3,221.50	SW	**1.55**	11.55
**C4**	**90**	**60**	296,219.30	NE	-122.86	-112.86	8,719.67	NE	-3.07	6.93
**BC**	**80**	**98**	33,461.72	SW	**26.62**	36.62	24,516.17	SW	**19.21**	29.21
**H**	**90**	**98**	nLRDT Dominant	SE	**28.32**	38.32	nLRDT Dominant	SE	**20.90**	30.90
	**Scenario 2: Tongue swab + 1-day nLRDT healthcare visit**									
			**Societal Perspective**				**Provider Perspective**			
	**nLRDT Sensitivity, %**	**nLRDT Specificity, %**	**ICER (nLRDT vs. Xpert)**	**Cost-Effectiveness Plane Quadrant**	**NMB, US$**	**Threshold cost-effective price, US$**	**ICER (nLRDT vs. Xpert)**	**Cost-Effectiveness Plane Quadrant**	**NMB, US$**	**Threshold cost-effective price, US$**
**C1**	**60**	**90**	4,456.40	SW	**10.29**	20.29	6,793.77	SW	**18.06**	28.06
**C2**	**70**	**80**	nLRDT Dominated	NW	-27.80	-17.80	7,834.60	SW	**13.44**	23.44
**C3**	**80**	**70**	nLRDT Dominated	NW	-65.89	-55.89	12,003.00	SW	**8.83**	18.83
**C4**	**90**	**60**	250,902.20	NE	-103.98	-93.98	nLRDT Dominant	SE	**4.21**	14.21
**BC**	**70**	**98**	22,469.38	SW	**43.81**	53.81	13,305.03	SW	**24.79**	34.79
**H**	**90**	**98**	nLRDT Dominant	SE	**47.20**	57.20	nLRDT Dominant	SE	**28.18**	38.18

In the north-east quadrant ICERs below the WTP threshold indicate the nLRDT is cost-effective, whereas in the south-west quadrant ICERs greater than the WTP threshold indicate the nLRDT is cost-effective. NMBs were calculated using the willingness-to-pay estimate of $1357/QALY and based on the nLRDT estimated cost of $10 per unit. Threshold cost-effectiveness prices were calculated by adding net NMB to the assumed $10 cost of the nLRDT. Costs were adjusted for inflation and expressed in 2024 US dollars. Positive NMB values indicate the nLRDT is cost-effective whilst negative NMB values indicate the nLRDT is not cost-effective. Scenario 1 is the standard scenario. Scenario 2 is the scenario where nLRDT uses tongue swab samples, enabling completion of the diagnostic process within one day. Net monetary benefit is expressed in 2024 US dollars. Abbreviations: C1-C4, conservative cases 1–4; BC, base case; H, high performance case; NMB, net monetary benefit; ICER, incremental cost-effectiveness ratio.

### Univariate deterministic sensitivity analysis

**Fig 2:**
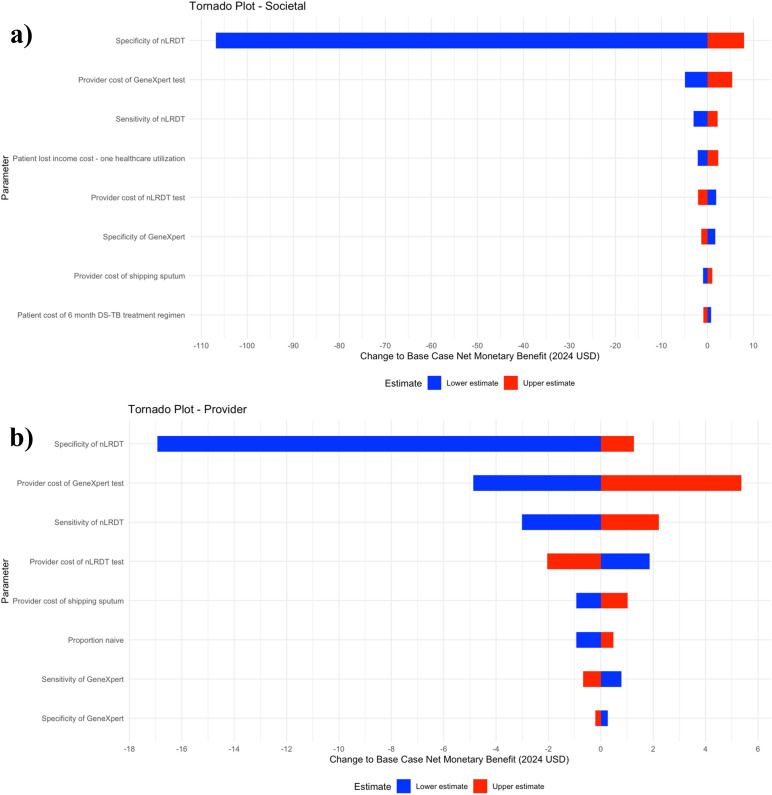
Tornado plots showing the eight parameters whose uncertainty most affects cost-effectiveness, from both (a) Societal and (b) Provider perspectives. Lower and upper estimates are the 2.5^th^ percentile and 97.5^th^ percentile values taken from each parameter’s uncertainty distribution. Net monetary benefit is expressed in 2024 US dollars.

Fig 2 shows that the societal and provider perspectives share several key parameters affecting cost-effectiveness, though their relative importance varies. nLRDT specificity, provider cost of Xpert, and nLRDT sensitivity cause the biggest changes relative to the base case NMB when analysed at their lower and upper estimates, with specificity being the most influential driver. However, the relative importance of the provider cost of Xpert and nLRDT sensitivity decreases in the provider perspective compared to the societal perspective. Unique to the societal perspective diagram are parameters such as patient lost income cost and patient cost of the DS-TB treatment regimen, as these are not considered in the provider perspective. Conversely, the provider perspective places greater emphasis on the proportion of naïve patients and the provider costs of nLRDT and sputum sample transportation.

### Multivariate deterministic sensitivity analysis

**Fig 3:**
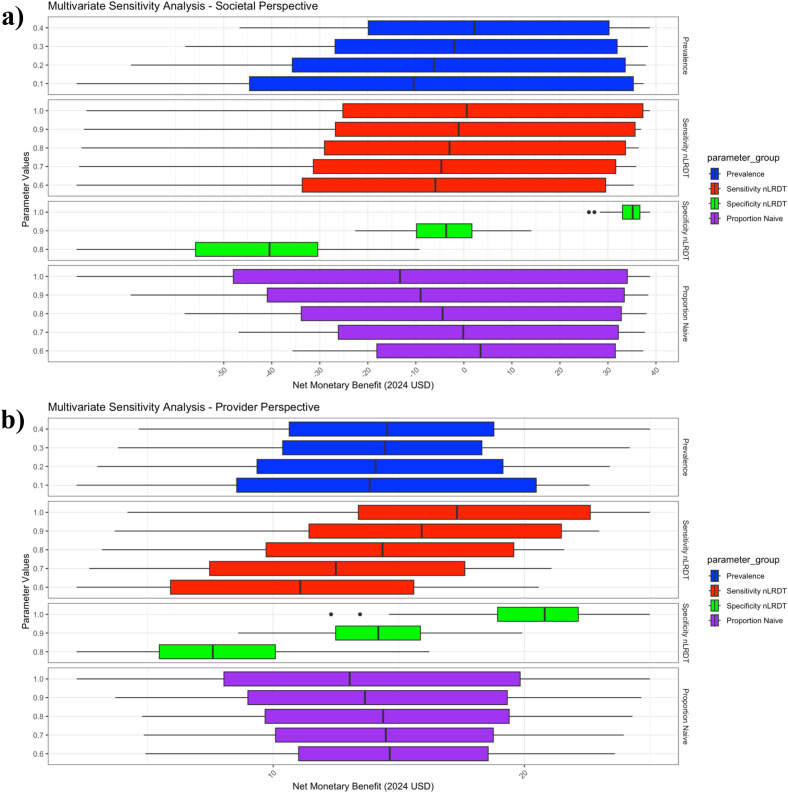
Multivariate sensitivity analysis of four model parameters on the net monetary benefit of the nLRDT, from both (a) societal and (b) provider perspectives. The boxplots illustrate the median, interquartile range, and variability of net monetary benefit for all simulated parameter sets. Each parameter set consists of a combination of prevalence, proportion of the population that is treatment-naïve and sensitivity and specificity of the nLRDT. The chosen ranges for each parameter are displayed on the y-axis of the graph. The bar whiskers extend to 1.5 times the interquartile range, and the dots represent outliers beyond this range. Net monetary benefit is expressed in 2024 US dollars.

Fig 3 illustrates that from the societal perspective, nLRDT specificity, prevalence and proportion naïve are all important covariates of the NMB of the nLRDT, with specificity being the most critical. When specificity is perfect, the NMB shows little variation when accounting for all combinations of the other parameter values within their chosen ranges and remains significantly positive. In contrast, with a specificity of 80%, the NMB is negative across all other parameter combinations, with a median NMB of around $-40, compared to a median of around $35 in the perfect specificity scenario.

From the provider perspective, the NMB is less sensitive to prevalence and the proportion naïve, and more sensitive to nLRDT sensitivity, with specificity remaining the key covariate. However, NMB is less sensitive to specificity in the provider perspective than in the societal perspective. For all three considered specificity values, NMB remains positive, ranging from a median of approximately $7 when specificity is 80% to just over $20 with perfect specificity. Additional multivariate sensitivity analyses are displayed in Fig A in S1 File.

### Probabilistic sensitivity analysis

**Fig 4:**
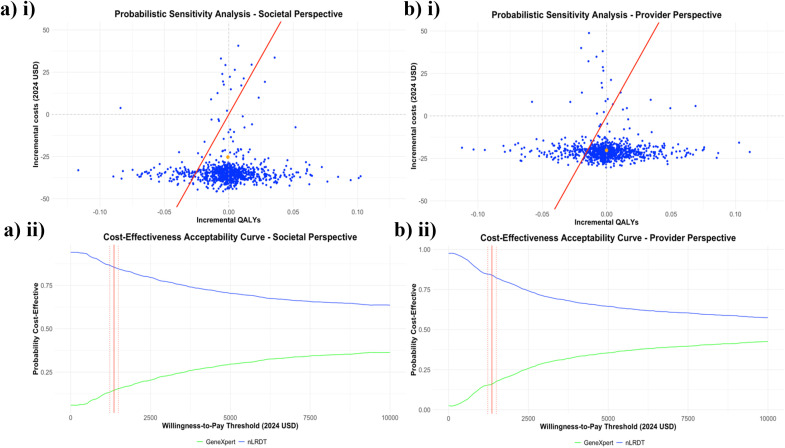
Results of the probabilistic sensitivity analysis. (i) Scatter plots on the cost-effectiveness plan and (ii) cost-effectiveness acceptability curves from both the (a) societal and (b) provider perspectives. 1000 Monte Carlo model simulations were conducted, the orange dot indicates the mean result of the simulations. In (ii) the vertical red line shows the estimated willingness-to-pay threshold of $1357/QALY, along with a 10% uncertainty (dotted red lines) either side of this value.

Fig 4 shows that, from both perspectives, most of the incremental cost-effectiveness simulations, as well as the mean result, lie below the x-axis of the scatter plot, indicating that the nLRDT is likely cost-saving. Furthermore, from both perspectives, there is no WTP threshold at which less than 50% of the probabilistic ICERs fall below the WTP threshold. Both graphs demonstrate convergence at an nLRDT probability of cost-effectiveness greater than 50% for very high thresholds (>$10,000/QALY). Furthermore, the probability of the nLRDT intervention being cost-effective is greater under the societal perspective than the provider perspective, at all evaluated thresholds.

## Interpretation

The study’s major findings are two-fold. Firstly, the proposed integration of the nLRDT as an initial diagnostic intervention in rural Philippine settings was estimated to be cost-effective compared to the current standard of care using Xpert [24], using the estimated WTP threshold of $1357/QALY [32], under base case test performance assumptions and from both societal and provider perspectives. Notably, the approach yields a greater estimated NMB from the societal perspective. Secondly, nLRDT specificity is the most critical factor in determining cost-effectiveness under both perspectives, in line with San Miguel et al. [42], who concluded that test specificity is a ‘very important factor’ of economic evaluations comparing diagnostics, as explained below. The scenario analyses, UVA and MVA, showed nLRDT specificity held more importance as a determinant of cost-effectiveness from the societal perspective. Further investigation of the terminal node outcomes under different test performance assumptions revealed this was due to the number of ‘false positives’, a function of specificity. When nLRDT specificity is low, many patients fall into the ‘false positive’ category. The estimated patient cost associated with a false positive (~$544) diagnosis is far higher than the provider cost (~$103) [26,37]. Thus, poor specificity makes the nLRDT appear more cost-effective from the provider perspective. As nLRDT specificity increases (e.g., to 98% in the base case), false positive cohort size diminishes, and the nLRDT cost-savings become greater from the societal perspective.

A key strength of this study is the use of reliable, nationwide data sources to parameterise the model: patient cost data from a national TB patient cost survey conducted by the Philippines NTP [26], provider unit cost data from a substantial random sampling study of 28 health facilities across the Philippines [37], and the latest national drug resistance survey conducted by the Philippine National Tuberculosis Reference Laboratory [29]. Another strength is the clinician collaboration, ensuring the model was appropriately contextualised to the Philippines and reflected a feasible and pragmatic use case for the nLRDT. Furthermore, the extensive sensitivity analyses and coverage of multiple scenarios enhance the future relevance of our results, particularly as certainty regarding the nLRDT clinical performance increases following large-scale trials. Evaluating the stability and robustness of findings across plausible parameter ranges provides decision-makers a clear understanding of how parameter uncertainties impact cost-effectiveness of the nLRDT. Moreover, the results are relevant to various stakeholders. For example, the MVA may interest local policymakers, as evaluated parameters like prevalence and the proportion of treatment-naive patients will likely differ across regions, with Fig 3 highlighting the influence of specificity in determining cost-effectiveness. Additionally, the simplicity of the Markov model is a strength, straightforwardly capturing patients’ capacity to benefit from different treatment regimens and aligning with the study focus of comparing diagnostic interventions.

However, this study has several limitations. Firstly, it considered only one hypothetical clinical pathway for nLRDT incorporation without exploring structural optimisation for a more cost-effective use-case. Additionally, assuming a full day’s income loss for healthcare visits may overestimate the patient’s cost. In Fig 2, the UVA showed that a lower estimate of this parameter led to decreased cost-effectiveness estimates in the societal perspective. The UVA identified this parameter as the fourth most influential driver of cost-effectiveness from the societal perspective, suggesting alternative estimates could have a substantial impact on results.

Fig 2 also highlighted the importance of sputum transportation costs as a driver of cost-effectiveness, with greater relative importance under the provider perspective. Due to an absence of data, expert opinion was sought for an estimate though more robust data would have been preferable. Finally, the modelling package did not easily allow the decision tree to be comprehensively combined with the Markov models, meaning the impact of treatment outcome probabilities on cost-effectiveness results could not be directly investigated. However, as ‘Model QALYs’ was not one of the key parameters in Fig 2, this suggests that treatment efficacy did not particularly favour one diagnostic over another.

Another potential limitation is the decision not to model secondary infections. Including re-infections should not impact the perceived nLRDT cost-effectiveness, as re-infection rates would be consistent across both comparator branches (re-infection should not depend on the initial diagnostic intervention). However, secondary infections would affect cost-effectiveness. Among the four diagnostic outcomes (true/false, positive/negative), secondary infections can only be caused by true positives and false negatives, with the latter being particularly concerning as they may unknowingly spread PTB [46]. Although nLRDT promises quicker results than Xpert, reducing the scope for secondary infections between initial PHC presentation and diagnosis, its lower sensitivity will result in more false negatives, likely reducing its cost-effectiveness. Future research should explore the impact of secondary infections on nLRDT cost-effectiveness.

For policymakers, several unmodelled nLRDT benefits should be considered alongside health economic evaluation. Firstly, its minimal resource requirements and ability to be conducted in rural decentralised settings suggest that existing healthcare infrastructure likely already meets most of its demands, enabling a quick and economical nationwide scale-up of the test. Secondly, tongue swabs eliminate the need for potential invasive procedures on children who struggle with sputum collection [15], making TB testing more accessible and less costly, as well as less stigmatising and with better implications for infection control [47]. Finally, as the intervention can test a greater number of people for PTB closer to home, it will promote care-seeking among patients. The projected greater uptake of testing will result in more patients with PTB being treated, thus reducing onward transmission and increasing overall population health outcomes. Furthermore, the nLRDT would be useful in non-rural health centres that do not have Xpert currently available.

While these results are Philippines-specific, they may be informative for other LMICs with similar TB profiles, such as comparable resistance levels and prevalence among presumptive PTB cases. The evidence suggests that the nLRDT has the capacity to be cost-effective in these settings, as has been estimated for the Philippines. We recommend further setting-specific cost-effectiveness research once more target settings have been established and there is less uncertainty surrounding key model parameters, like nLRDT sensitivity and specificity, following completion of large-scale clinical trials.

## Supporting information

S1 File **Table A**. Model assumptions and their justifications. **Table B**. Model parameters and their assigned probability distributions. **Fig A**: Multivariate sensitivity analysis, repeated under fixed nLRDT specificity valu.s a) Societal perspective b) Provider perspective.
(DOCX)
