# Supplementary material for: Early cost-effectiveness analysis of a novel rapid diagnostic test for tuberculosis in rural Philippine settings
Source: PLOS Glob Public Health. 2025 Oct 28;5(10):e0005364. doi: 10.1371/journal.pgph.0005364 (PMC12561941; doi:10.1371/journal.pgph.0005364)
Supplement: S1 File — Table A. Model assumptions and their justifications. Table B. Model parameters and their assigned probability distributions. Fig A: Multivariate sensitivity analysis, repeated under fixed nLRDT specificity valu.s a) Societal perspective b) Provider perspective. (DOCX) [file pgph.0005364.s001.docx]

# Supplementary materials

## Model assumptions

Table A**:** Model assumptions and their justifications.

| **No.** | **Model Assumption** | **Justification** |
| --- | --- | --- |
| 1. | Base case nLRDT sensitivity and specificity were assumed to be 80% and 98%, respectively. | Based on the WHO Product Profile’s ‘minimal requirements’ for a ‘rapid sputum-based test for detecting TB in decentralized settings’,(10) alongside expert opinion for most likely values. |
| 2. | Base case nLRDT cost was assumed to be $10. | This estimate encapsulates unit staff (including training), overhead, consumables (based on manufacturer’s production and supply chain estimates) and capital costs.  Furthermore, ‘cost-effective’ nLRDT healthcare provider prices (calculated for the estimated WTP threshold) are presented in ***Results*** for various scenarios. This may be of interest to policymakers and stakeholders financing the wider project. |
| 3. | The Xpert rifampicin resistance (RR) test was assumed to have 100% sensitivity and specificity, correctly identifying all patients’ true resistance status. | 1) The high reported sensitivity (95%) and specificity (98%) values,(11) coupled with 2) clinicians’ use of algorithms to assess MDR-TB risk (e.g., previous treatment or contact with an MDR-TB patient), making resistance misdiagnosis ‘uncommon’, according to Dr. Ortal-Cruz. |
| 4. | There was assumed to be no resistance among treatment-naïve patients. | Pragmatic assumption made for model simplicity, supported by low reported (1.8%) resistance rates in this cohort.(2) |
| 5 | All RR patients were assumed to also be resistant to isoniazid and were therefore classified MDR-TB. | High rates of isoniazid resistance (~70%) found in RR isolates in the Philippines.(2) |
| 6. | There were assumed to be no re-infections or secondary infections. | By focusing on the initial diagnosis and treatment phase, the complexities of modelling re-infections and secondary infections are avoided, thereby maintaining attention on the study aim. |
| 7. | 1) The assumed treatment for all DS-TB patients is 2HRZE/4HR – 2 months of isoniazid, rifampicin, pyrazinamide, and ethambutol followed by 4 months of isoniazid and rifampicin.  2) The assumed treatment for all MDR-TB patients was a 9-month standard short oral regimen | 1) This is recommended for all DS-TB PTB patients.(12)  2) 99% of patients, who are at least resistant to rifampicin, in the Philippines, receive the standard short oral regimen.(13) |
| 8. | All false positives were assumed to receive DS-TB treatment regimens. | Diagnosis of resistance will use discussed algorithms to determine MDR-TB risk. Given this cohort do not have PTB, it is unlikely they will be at high risk of MDR-TB. |
| 9. | Treatment begins immediately following diagnosis. We assume full compliance from healthcare workers using nLRDT-guided clinical management and from patients adhering to national guideline treatment plans. | Pragmatic assumption for model simplicity but is supported by the Philippines’ strong and extensive network of WHO-endorsed Directly Observed Treatment, Short-Course (DOTS) facilities, which guarantee that patients adhere to their regimen,(14) improving cure rates and preventing medication resistance.(15) |
| 10. | There were assumed to be no adverse drug reactions. | Following Chitpim et al.(3) |
| 11. | Patients with a false-negative result were assumed to delay seeking TB care for at least one month, resulting in increased treatment death rates. | In the absence of studies in the Philippines modelling the clinical pathway of false negatives, a Southwest Ethiopia study reported TB treatment mortality rates more than doubled when treatment delays exceeded 30 days.(16) |
| 12. | 1) There was assumed to be no patient direct medical costs between presenting with presumptive TB and receiving a diagnosis.  2) All costs before initial presentation were ignored. | 1) Supported by widespread PhilHealth coverage which covers diagnosis costs.(7)  2) These costs should be the same for both interventions and thus do not contribute our cost-effectiveness analysis. |
| 13. | One healthcare visit was assumed to result in one day’s income loss for the patient. | While various studies estimate patient income loss from symptom onset,(8,17) this study focuses on costs incurred from the first healthcare visit onward, in line with the study aim. Thus, a simple estimate derived from average daily GDP per capita is used. |
| 14. | There were assumed to be no further investigations beyond an Xpert test. | Although additional investigations (e.g., repeat Xpert or culture tests) may occur, particularly in low-risk resistance patients who test positive for MDR-TB, this was not considered in the model for simplicity, however clinical expert opinion (Dr. Ortal Cruz) confirmed this would only apply to a small subset of the total patient population (given 12% of patients are treatment experienced, and 16% of those resistant). |
| 15. | There was assumed to be no extensive drug-resistant TB. | Despite other studies incorporating XDR-TB,(3) the small proportion of XDR-TB patients (0% of MTB-positive by Xpert patients) in the Philippines did not warrant adding this complexity to the ‘treatment’ aspect of the model,(2) given the study aim of intervention comparison. |
| 16. | The average age of a patient entering the model is assumed to be 40. | The Philippines national TB survey reported an average TB patient age of 40.2 years.(7) |
| 17. | Xpert was assumed as the comparator in all cases, despite smear microscopy remaining the primary diagnostic tool in some regions (). | Pragmatic assumption for simplicity, aligning with the 91% reduction in smear microscopy diagnostics between 2017-2022 and the rapid nationwide uptake of Xpert in recent years following the 2019 NTP goal for all PTB cases to be tested with Xpert.(3,7) |
| 18. | Xpert diagnosis requires three healthcare visits on separate days: an initial physician assessment, sputum collection and a follow-up for results and treatment initiation. Although two-day completion is possible, most health centres have an early morning cut-off for sputum collection (around 9 AM), necessitating an additional visit.  Diagnosis with the nLRDT requires two healthcare visits on separate days: an initial physician assessment and a combined visit for the nLRDT test and results/treatment discussion, the latter completed in one day. | Dr. Ortal-Cruz discussions regarding current clinical pathway and theoretical integration of the nLRDT into target setting. |

Abbreviations: DS-TB, drug sensitive tuberculosis; MDR-TB, multidrug-resistant tuberculosis; XDR-TB, extensive drug-resistant tuberculosis; RR, rifampicin resistance; PTB, pulmonary tuberculosis; WTP, willingness-to-pay; NTPMP6, National TB Program Manual of Procedures 6^th^ edition.

## Model parameters

Table B. Model parameters and their assigned probability distributions

| **Parameter** | **Distribution** | **Mean** | **Standard Error** | **Shape** | **Scale** | **Source** | **Notes** |
| --- | --- | --- | --- | --- | --- | --- | --- |
| Prevalence of PTB among suspected PTB patients | Beta | 0.3 | 0.03 | 70 | 165.7 | (1) |  |
| Proportion of population that is treatment-naïve | Beta | 0.88 | 0.09 | 11.41 | 1.6 | (2) | Proportion of treatment-experienced deduced from this. |
| Proportion of treatment-experienced PTB patients with rifampicin resistance | Beta | 0.17 | 0.02 | 83.34 | 421.73 | (2) | Along with assumption that there is no resistance among treatment-naïve (see **Table 1.4 in source**), a general population pooled resistance proportion was estimated from this. |
| Sensitivity of Xpert | Beta | 0.88 | 0.02 | 222.24 | 30.31 | (3) | Probability distribution sourced from literature. |
| Specificity of Xpert | Beta | 0.99 | 0.003 | 1505.07 | 15.2 | (3) | Probability distribution sourced from literature. |
| Sensitivity of nLRDT | Beta | 0.80 | 0.08 | 19.2 | 4.8 | Expert opinion |  |
| Specificity of nLRDT | Beta | 0.98 | 0.1 | 1.02 | 0.02 | Expert opinion |  |
| Proportion of nLRDT negatives who receive further investigation based on convincing symptoms | Beta | 0.15 | 0.02 | 84.85 | 480.82 | Expert opinion | A function of prevalence and sensitivity. Upper bound of this variable assumed to be prevalence (mean of 0.297), and lower bound is zero. Therefore, value halfway between chosen as mean. |
| **Test Costs – Provider** | | | | | | | |
| Provider cost of an outpatient diagnostic visit, US$ | Gamma | 4.11 | 0.41 | 100 | 0.04 | (4) | Average of bottom-up and top-down costs. |
| Provider cost an outpatient treatment visit, US$ | Gamma | 3.29 | 0.33 | 100 | 0.03 | (4) | Average of bottom-up and top-down costs. |
| Provider cost of a sputum collection, US$ | Gamma | 7.28 | 0.73 | 100 | 0.07 | (4) | Average of bottom-up and top-down costs. |
| Provider cost of shipping sputum, US$ | Gamma | 5.00 | 0.5 | 100 | 0.05 | Expert opinion | Based on recommendations from the NTPMP6, which advises the use of triple packaging and cold packs without the need for sophisticated equipment. |
| Provider cost of Xpert test, US$ | Gamma | 23.95 | 2.61 | 100 | 0.26 | (5,6) | Average of bottom-up and top-down costs. Removed $2.01 from cited value, due to cartridge price reduction occurring in 2023. This value includes staff, consumables, overheads and capital cost. It is predominantly comprised of consumables. |
| Provider cost of nLRDT test, US$ | Gamma | 10.00 | 1.00 | 100 | 0.10 | Cost-breakdown provided by manufacturer + expert opinion | Similar to nLRDT, staff (including training), consumables, overheads and capital costs considered in estimation. |
| **Test Costs - Patient** | | | | | | | |
| Food, accommodation & travel - One Visit, US$ | Gamma | 0.29 | 0.03 | 100 | 0.003 | (5) | Post-diagnosis accommodation, food, and travel costs were divided by the mean number of post-diagnosis healthcare visits to get a proxy estimate for the cost per visit during the diagnosis process. |
| Income loss - One Visit, US$ | Gamma | 11.31 | 1.13 | 100 | 0.11 | (6) | The Philippines average GDP per capita per day, 2024. |
| **Treatment Costs - Provider** | | | | | | | |
| Provider cost of DS-TB treatment, US$ | Gamma | 102.54 | 10.25 | 100 | 1.03 | (4) | Average of bottom-up and top-down costs. |
| **Parameter** | **Distribution** | **Mean** | **Standard Error** | **Shape** | **Scale** | **Source** | **Comments** |
| Provider cost of MDR-TB treatment, US$ | Gamma | 1439.48 | 143.95 | 100 | 14.4 | (4) | Average of bottom-up and top-down costs. |
| **Treatment Costs – Patient** | | | | | | | |
| Rural patient cost of DS-TB treatment, US$ | Gamma | 544.27 | 54.43 | 100 | 5.44 | (7) | Patient costs were stratified by rural and urban patients. First, proportion of total household lost hours specific to the patient, that were not pre-disease was calculated, and multiplied by total household income loss to get post-diagnosis, patient income loss. |
|  |  |  |  |  |  |  | This was added to post-diagnosis direct medical costs (drug pick-up, directly observed therapy, follow-up & hospitalization costs). This was added to post-diagnosis direct non-medical costs (travel, food, accommodation & nutrition supplement costs). |
| Patient cost of MDR-TB treatment, US$ | Gamma | 2999.50 | 299.95 | 100 | 30 | (7) | Patient costs were not stratified by urban and rural patients for MDR-TB patients. Same calculation as above. |
| **Treatment Outcomes - DS-TB (6-month proportions)** | | | | | | | |
| Success | Assumed constant in Markov model. | 0.97 | Assumed constant in Markov model. | | | (8) |  |
| Death |  | 0.03 |  |  |  | (8) |  |
| Treatment failure (DS-TB -> MDR-TB) |  | 0.003 |  |  |  | (8) |  |
| **Treatment Outcomes - MDR-TB (9-month proportions)** | | | | | | | |
| Success | Assumed constant in Markov model. | 0.85 | Assumed constant in Markov model. | | | (8) |  |
| Death |  | 0.15 |  |  |  | (8) |  |
| **Treatment Outcomes - False Negatives DS-TB (6-month proportions)** | | | | | | | |
| Success | Assumed constant in Markov model. | 0.96 | Assumed constant in Markov model. | | | (8) |  |
| Death |  | 0.04 |  |  |  | (8) | Assumed 150% of true positive death rate (see **Table 1.11 in source**) |
| Treatment failure (DS-TB -> MDR-TB) |  | 0.003 |  |  |  | (8) |  |
| **Treatment Outcomes - False Negatives MDR-TB (9-month proportions)** | | | | | | | |
| Success | Assumed constant in Markov model. | 0.77 | Assumed constant in Markov model. | | | (8) |  |
| Death |  | 0.23 |  |  |  | (8) |  |
| **Markov Model Health State Utilities** | | | | | | | |
| Ongoing DS-TB treatment utility | Assumed constant in Markov model. | 0.69 | Assumed constant in Markov model. | | | (9) |  |
| Ongoing MDR-TB treatment utility |  | 0.51 |  |  |  | (9) | Philippines specific data could not be obtained, data from Thailand used. |
| Cured |  | 0.88 |  |  |  | (9) |  |
| **Markov Model Outcomes** | | | | | | | |
| Lifetime QALYs per DS-TB patient | Gamma | 8.44 | 0.84 | 100 | 0.08 |  |  |
| Lifetime QALYs per MDR-TB patient | Gamma | 7.70 | 0.77 | 100 | 0.08 |  | These were varied collectively in the sensitivity analyses. * |
| Lifetime QALYs per false negative DS-TB patient | Gamma | 8.38 | 0.84 | 100 | 0.09 |  |  |
| **Parameter** | **Distribution** | **Mean** | **Standard Error** | **Shape** | **Scale** | **Source** | **Comments** |
| Lifetime QALYs per false negative MDR-TB patient | Gamma | 7.12 | 0.71 | 100 | 0.07 |  | * |
| Lifetime costs per DS-TB patient, US$ | Gamma | 1.38 | 0.14 | 100 | 0.01 |  | This is an average per patient cost of failing treatment and receiving MDR-TB treatment. This cost only exists for DS-TB patients, as MDR-TB patients die if they fail treatment. |
| Lifetime costs (from treatment failure) per false negative DS-TB patient, US$ | Gamma | 1.49 | 0.15 | 100 | 0.02 |  | This is an average per patient cost of failing treatment and receiving MDR-TB treatment. This cost only exists for DS-TB patients, as MDR-TB patients die if they fail treatment. |

Values were reported to two decimal places; one significant figure for very small values. Costs are expressed in 2024 US dollars. Abbreviations: PTB, pulmonary tuberculosis; DS-TB, drug sensitive tuberculosis; MDR-TB, multidrug-resistant tuberculosis; QALY, quality adjusted life year; NTPMP6, National TB Program Manual of Procedures 6th edition.

## Additional multivariate analyses

In Fig A(a)**,** when nLRDT specificity is low (0.8), prevalence and proportion naïve are the most critical covariates for determining the NMB. As specificity increases (to 0.9 and 1), nLRDT sensitivity gains greater relative importance compared to the other parameters in determining NMB. Fig A(b) shows that, from the provider perspective, the NMB is most sensitive to nLRDT sensitivity across all three fixed specificity values. Furthermore, under both perspectives, NMB increases with prevalence at specificity values of 0.8 and 0.9 but decreases with prevalence in the perfect specificity case. Similarly, from both perspectives, NMB decreases with proportion naïve at specificities of 0.8 and 0.9 but increases with proportion naïve in the perfect specificity case.

Fig A**:** Multivariate sensitivity analysis, repeated under fixed nLRDT specificity values a) Societal perspective b) Provider perspective

**(a)**

***
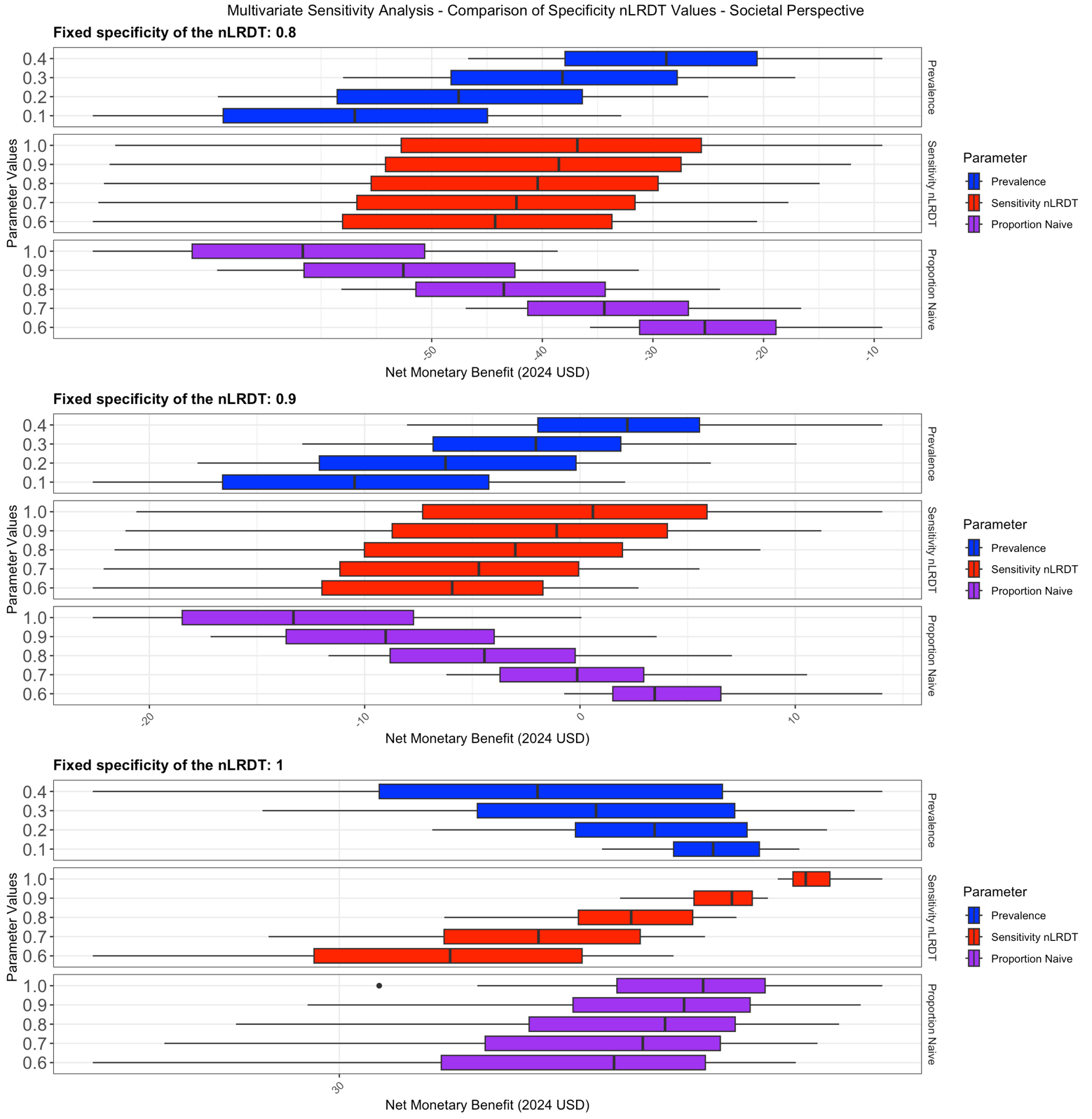
***

**(b)**

***
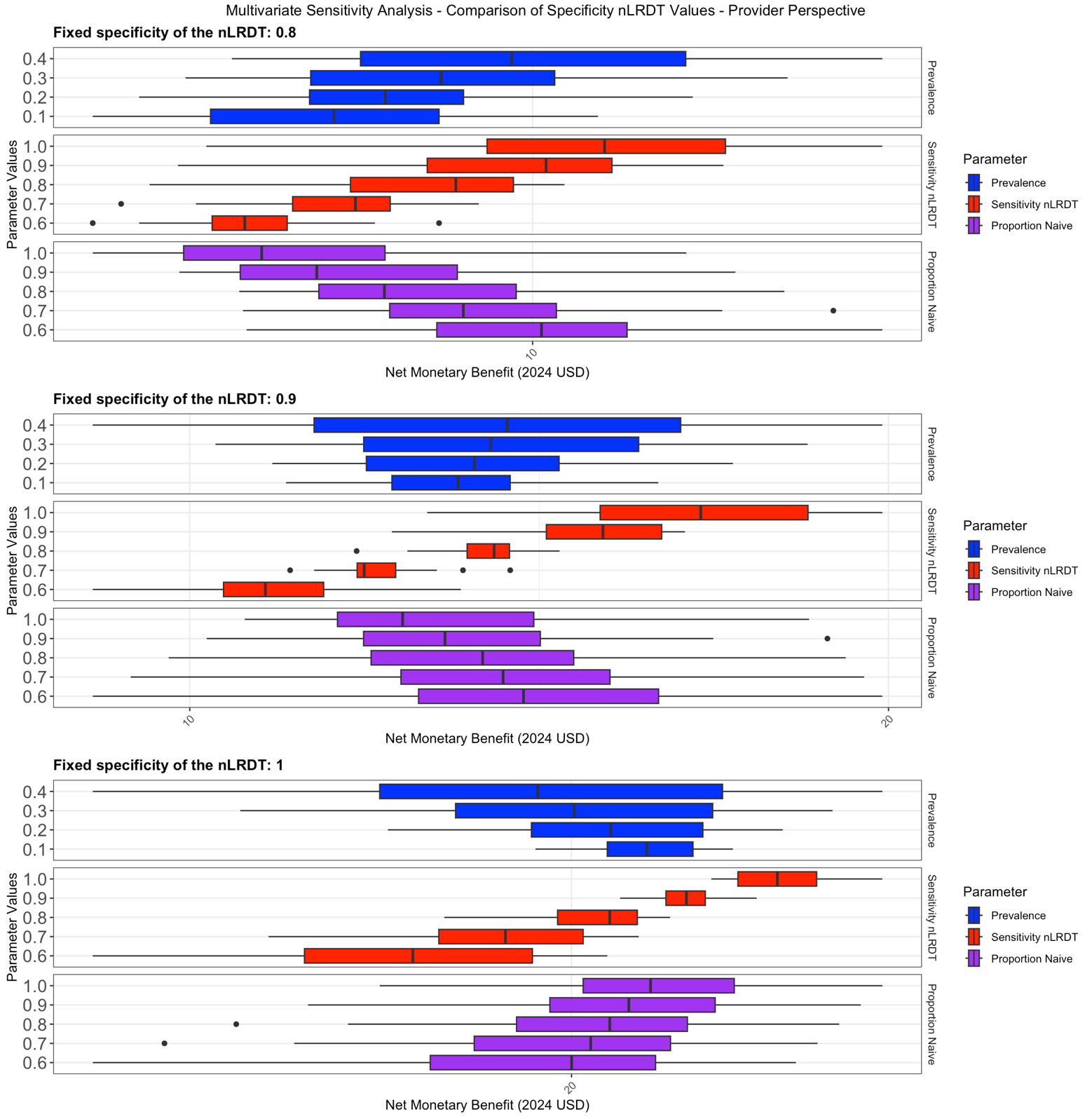
***

The boxplots illustrate the median, interquartile range, and variability of the net monetary benefit for all simulated parameter sets. Each parameter set consists of a combination of prevalence, proportion of the population that is treatment-naïve and sensitivity of the nLRDT. The chosen ranges for each parameter are displayed on the y-axis of the graph. The bar whiskers extend to 1.5 times the interquartile range, and the dots represent outliers beyond this range. Net monetary benefit is expressed in 2024 US dollars.

# List of legends

- Table A: Model assumptions and their justifications
- Table B. Model parameters and their assigned probability distributions
- Fig A: Multivariate sensitivity analysis, repeated under fixed nLRDT specificity values a) Societal perspective b) Provider perspective

# References

1. Vianzon R, Garfin AMC, Lagos A, Belen R. The tuberculosis profile of the Philippines, 2003-2011: advancing DOTS and beyond. Western Pac Surveill Response J. 2013;4(2):11–6.

2. Ama MCG, Garfin AMCG, Basilio RP, Dayrit-Halum CB, Theng Ng Y, Butcon J V, et al. High Burden of Isoniazid Resistance - Findings from the 2018 Philippine Tuberculosis Drug Resistance Survey. 2024;

3. Chitpim N, Jittikoon J, Udomsinprasert W, Mahasirimongkol S, Chaikledkaew U. Cost-Utility Analysis of Molecular Testing for Tuberculosis Diagnosis in Suspected Pulmonary Tuberculosis in Thailand. Clinicoecon Outcomes Res. 2022;14:61–73.

4. Capeding TPJ, Rosa JD, Lam H, Gaviola DG, Garfin AMC, Hontiveros C, et al. Cost of TB prevention and treatment in the Philippines in 2017. Int J Tuberc Lung Dis. 2022 May;26(5):392–8.

5. Yamanaka T, Castro MC, Ferrer JP, Solon JA, Cox SE, Laurence Y V, et al. Costs incurred by people with co-morbid tuberculosis and diabetes and their households in the Philippines. PLoS One [Internet]. 2024 Jan 25;19(1):e0297342. Available from: https://doi.org/10.1371/journal.pone.0297342

6. International Monetary Fund. IMF DataMapper - Philippines [Internet]. 2024 [cited 2024 Aug 23]. Available from: https://www.imf.org/external/datamapper/profile/PHL

7. Florentino JL, Arao RML, Garfin AMC, Gaviola DMG, Tan CR, Yadav RP, et al. Expansion of social protection is necessary towards zero catastrophic costs due to TB: The first national TB patient cost survey in the Philippines. PLoS One. 2022;17(2):e0264689.

8. World Health Organization. Global Tuberculosis Report 2023 - Tuberculosis Data [Internet]. 2023 [cited 2024 Aug 23]. Available from: https://www.who.int/teams/global-tuberculosis-programme/data

9. Kittikraisak W, Kingkaew P, Teerawattananon Y, Yothasamut J, Natesuwan S, Manosuthi W, et al. Health related quality of life among patients with tuberculosis and HIV in Thailand. PLoS One [Internet]. 2012;7(1):e29775. Available from: http://ovidsp.ovid.com/ovidweb.cgi?T=JS&PAGE=reference&D=med9&NEWS=N&AN=22253777

10. Gupta-Wright A, den Boon S, MacLean EL, Cirillo D, Cobelens F, Gillespie SH, et al. Target product profiles: tests for tuberculosis treatment monitoring and optimization. Bull World Health Organ. 2023 Nov;101(11):730–7.

11. Ghebrekristos YT, Beylis N, Centner CM, Venter R, Derendinger B, Tshivhula H, et al. Xpert MTB/RIF Ultra on contaminated liquid cultures for tuberculosis and rifampicin-resistance detection: a diagnostic accuracy evaluation. Lancet Microbe. 2023 Oct;4(10):e822–9.

12. Republic of the Philippines National Tuberculosis Control Program. NTP Manual of Procedures 6th Edition [Internet]. 2021 [cited 2024 Aug 23]. Available from: https://ntp.doh.gov.ph/download/ntp-mop-6th-edition/

13. Sweeney S, Berry C, Kazounis E, Motta I, Vassall A, Dodd M, et al. Cost-effectiveness of short, oral treatment regimens for rifampicin resistant tuberculosis. PLOS Global Public Health [Internet]. 2022 Dec 7;2(12):e0001337. Available from: https://doi.org/10.1371/journal.pgph.0001337

14. Garfin C, Mantala M, Yadav R, Hanson CL, Osberg M, Hymoff A, et al. Using Patient Pathway Analysis to Design Patient-centered Referral Networks for Diagnosis and Treatment of Tuberculosis: The Case of the Philippines. J Infect Dis. 2017 Nov;216(suppl_7):S740–7.

15. Davies PDO. The role of DOTS in tuberculosis treatment and control. Am J Respir Med [Internet]. 2003;2(3):203–9. Available from: http://ovidsp.ovid.com/ovidweb.cgi?T=JS&PAGE=reference&D=med5&NEWS=N&AN=14720002

16. Asres A, Jerene D, Deressa W. Delays to treatment initiation is associated with tuberculosis treatment outcomes among patients on directly observed treatment short course in Southwest Ethiopia: a follow-up study. BMC Pulm Med [Internet]. 2018;18(1):64. Available from: https://doi.org/10.1186/s12890-018-0628-2

17. Republic of the Philippines Department of Health. About San Lazaro Hospital [Internet]. [cited 2024 Aug 23]. Available from: https://slh.doh.gov.ph/transparency/about-san-lazaro-hospital
